# Stapes surgery under local anaesthesia

**DOI:** 10.1308/003588413X13511609954932

**Published:** 2013-01

**Authors:** JA Lavy, HRF Powell

**Affiliations:** University College London Hospitals NHS Foundation Trust,UK

**Keywords:** Stapes surgery, Local anaesthesia

## Abstract

In the UK, stapes surgery is performed almost universally under general anaesthesia. In 1984 there was consensus that local anaesthesia should be the technique of choice in stapes surgery. Despite reports of successful use of local anaesthesia for middle ear surgery, this is still not widely accepted practice in the UK. We describe the senior author’s technique for local anaesthetic stapes surgery and present the hearing results for a series of 100 consecutive cases.

In the UK, stapes surgery is generally performed under general anaesthesia (GA). In other countries, including the US, local anaesthesia (LA) is often used for this procedure and, indeed, as early as 1984 there was consensus that local anaesthesia in stapes surgery should be the technique of choice.^1^ In 1997 Yung published his experience using local anaesthesia in middle ear surgery but he describes a take up rate of only 20%.^2^ It would appear that even now, 15 years later, use of LA is still the exception. The senior author has been performing stapes surgery under LA for over ten years and we present here the technique and outline the advantages.

## Methods

Almost all patients added to the waiting list for stapes surgery could have their operation performed under LA. In the senior author’s practice, the number insisting on GA is around 3%. The surgery and the technique are explained in full in the outpatient department when the patient is listed for operation. Many patients voice their concern about keeping still for a long period of time but can be reassured that, provided they ask first, they will be able to move their head to avoid neck stiffness. They are advised that the majority of people go home the same day and that simple analgesics are the only medications required postoperatively. They are encouraged to visit the senior author’s website and are given an email address for any further questions. Before leaving the clinic, a date for surgery is arranged.

At surgery, the patient and surgical field are prepared in the standard fashion with the head on a head ring. Aqueous povidone-iodine is the antiseptic preparation of choice and it is ensured that this fills the external auditory canal. The drapes are applied in such a way as to form a ‘tent’ so that with the operated ear uppermost the patient can look out from the drapes and even view the television monitor if he or she desires to watch the procedure.

**Figure 1:**
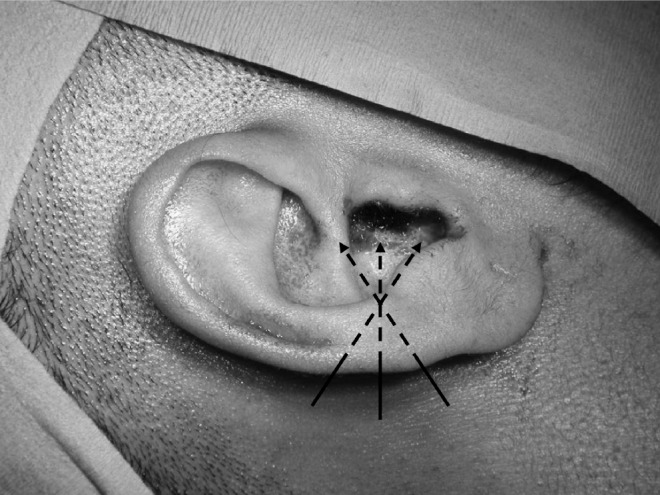
Dotted lines represent the path of the infiltrating needle once introduced into the postauricular crease

Prior to infiltration, the patient is warned that he or she may experience some temporary discomfort but that this will be short lived. Initially, the local anaesthetic needle is introduced in the postaural skin crease and advanced forward towards the posterior canal wall. Lidocaine hydrochloride 2% with adrenaline 1:80,000 is infiltrated very slowly. Faster infiltration is more uncomfortable. Further passes of the needle are made towards the roof and then the floor of the ear canal (Fig 1).

Infiltration in the canal is performed using a large Shea speculum just lateral to the junction of the hair bearing and the normal meatal skin. Again, this is infiltrated slowly to avoid undue discomfort and also ballooning of the deep meatal skin. The sites of infiltration are superiorly into the vascular strip, posteriorly (at nine o’clock for a right ear) and, finally, anteroinferiorly. The anterior injection is performed more medially in the canal than the other two, and should aim to be around the junction of the bony and cartilaginous portions of the canal. This injection enables the annulus to be elevated inferiorly without discomfort.

At the same time, intravenous sedation can be administered by an anaesthetist. A syringe driver with propofol is commonly used. Patients should be warned that the infusion might cause an uncomfortable sensation in the arm. The rate of administration should be tailored to the patient’s needs but it should be emphasised that it is better to aim for lighter rather than heavier sedation. With excessive sedation patients can become uncooperative. Many patients are suitable for surgery under LA without sedation but the option of sedation means that the technique is applicable to almost all adult patients.

**Table 1:** Mean pre and postoperative hearing results with the percentage of patients in each hearing grouping

ABG (0.5, 1, 2 and 3kHz)	Mean	≦0dB	1–10dB	11–20dB	>20dB
Preoperatively	31.0dB (SD: 10.0dB)	0%	0%	14%	86%
Postoperatively (comparison with postoperative BC)	7.7dB (SD: 4.5dB)	3%	83%	12%	2%
Postoperatively (comparison with preoperative BC)	4.7dB (SD: 4.9dB)	31%	58%	11%	0%

A single dose of 4mg intravenous dexamethasone is given as well as a vestibular sedative (cyclizine 50mg). The dexamethasone helps to reduce postoperative nausea and vestibular symptoms possibly due to an anti-inflammatory effect on the inner ear. For patients with particularly sensitive middle ear mucosa, a small pledget soaked in lidocaine hydrochloride 2% can be applied topically to good effect and then removed after a few minutes.

The surgical technique is a standard permeatal stapedotomy. Very rarely, owing to a narrow meatus and further narrowing as a result of infiltration, an endaural incision is required. The aural speculum is held in place using an adhesive OpSite^®^ dressing (Smith & Nephew, London, UK) as described by Kasbekar *et al*.^3^ This has the advantage that the patient is able to move his or her head without displacing the speculum. The senior author has been using a SMart^®^ (Olympus, Southend-on-Sea, UK) prosthesis since 2004. A potassium titanyl phosphate laser is used to divide the stapedius tendon and the posterior crus, and to perform a rosette stapedotomy. A 0.2mm diameter fibre is used with a 1W x 0.1 second single pulse setting.

In the presence of obliterative disease or a narrow niche, a Skeeter^®^ microdrill (Medtronic, Watford, UK) is used (0.6mm diamond paste burr) to widen the niche and blue-line the vestibule. Since 2007 clotted venous blood has been used to provide a seal around the prosthesis once in situ. Prior to this, no seal was used.

At the end of the procedure, the tympanic membrane is replaced and a clinical assessment of the hearing is performed (whisper voice at 50cm). This provides some feedback as to the coupling between the incus and the inner ear. Where the preoperative bone conduction thresholds are around 20dB or better, the clinical voice testing provides a simple subjective test of hearing improvement. If the whisper is not heard clearly, the tympanic membrane can be elevated and the prosthesis re-evaluated.

In patients with a mixed hearing loss, the senior author has found that the use of a handheld audiometer adds a degree of objectivity to this intraoperative test. Being activated by another member of the theatre staff, the audiometer is held 50cm from the ear and the patient is asked to identify when he or she hears a warble tone. The opposite ear is masked by occlusion on to the surgical head ring and also by tragal rubbing if necessary.

The ear is then packed with a strip of silastic sheeting laid across the flap and 0.5cm x 0.5cm pieces of ribbon gauze soaked with bismuth iodoform paraffin paste. The patient can return immediately to the ward and is usually discharged within a couple of hours. Simple analgesics are required in the first two or three days postoperatively. Antibiotic prophylaxis is not used routinely.

A discharge leaflet is given to the patients outlining what to expect over the next 2 weeks and a 24-hour telephone number is given for further queries. In addition, the senior author provides patients with his email address for any further communications during the first two weeks. Patients are advised to make contact urgently if they experience any sudden onset of severe dizziness, louder than normal tinnitus or pain.

Patients are reviewed in the outpatients department at two weeks. The dressings are removed from the ear and tuning fork tests performed. Patients are advised to keep the ear dry for a further two weeks. When the healing is not complete, a week of topical ciprofloxacin drops (ofloxacin) is prescribed three times daily. Audiometry with masked air and bone conduction thresholds including 3kHz is carried out at six weeks and six months postoperatively.

## Results

The senior author has been performing stapes surgery routinely under LA for over ten years. During that time, the technique has been adapted to achieve as high a percentage of day cases as possible. Presently, the senior author carries out between 80 and 90 stapes procedures (primary and revision) per year. The results of the most recent 100 primary LA procedures, which have full postoperative audiometry, are shown in more detail in Table 1.

## Discussion

The measured bone conduction may improve in successful stapes surgery due to the Carhart effect and therefore there are two sets of results. When looking at the postoperative air conduction compared with the preoperative bone conduction, 31 patients had an air-bone gap (ABG) of ≤0. This is known as overclosure. To give true results, the ABG in comparison with postoperative bone conduction at the same frequency is stated. To summarise the hearing results, 86% of patients started with an ABG of >20dB and 86% had a postoperative ABG of ≤10dB.

Of the 100 patients, 84 were discharged on the day of surgery. A further nine were deemed fit for discharge but elected to stay overnight for geographical reasons. The remaining seven patients stayed overnight owing to a combination of postoperative dysequilibrium and nausea. All seven were discharged the following morning.

Three of the patients in the series were undergoing second side surgery, having had their first ear done elsewhere under GA. All three patients commented that they preferred the local anaesthetic technique to their previous GA. Seven other patients have since or are due to have second ear surgery and none of them have requested a general anaesthetic. In all cases, the surgery was completed as expected and no procedure had to be abandoned owing to patient anxiety or discomfort. Over the same time period, there were three patients who chose to have their surgery under GA.

## Conclusions

Using this technique, local anaesthetic day case stapes surgery is a safe, well-tolerated procedure. With the additional availability of intravenous sedation, it is suitable for the majority of patients. The following points should be noted:

> The procedure must be explained fully and discussed in the clinic prior to listing the patient for surgery.
> Availability of an anaesthetist to administer intravenous sedation increases the number of suitable patients. Excess sedation, however, is counterproductive. (Less is more!)
> At the end of the procedure, the tympanic membrane is replaced to enable clinical assessment of the hearing prior to packing of the ear canal.
